# Predicting unplanned hospital readmission in palliative outpatients (PRePP) – study protocol of a longitudinal, prospective study to identify informal caregiver-related and structural predictors

**DOI:** 10.1186/s12904-022-00955-y

**Published:** 2022-05-02

**Authors:** Leopold Hentschel, André Wellesen, Luisa Christin Krause, Maria von Havranek, Michael Kramer, Beate Hornemann, Martin Bornhäuser, Ulrich Schuler, Katharina Schütte

**Affiliations:** 1 University Palliative Center, University Hospital Carl Gustav Carus, TU Dresden, Fetscherstraße 74, 01307 Dresden, Germany; 2 Department of Psychooncology, National Center for Tumor Diseases (NCT/UCC), Fetscherstraße 74, 01307 Dresden, Germany; 3 Core Unit Patient-Reported Outcomes, National Center for Tumor Diseases (NCT/UCC), Fetscherstraße 74, 01307 Dresden, Germany; 4 Medical Department I, University Hospital Carl Gustav Carus, TU Dresden, Fetscherstraße 74, 01307 Dresden, Germany; 5 National Center for Tumor Diseases (NCT/UCC), Fetscherstraße 74, 01307 Dresden, Germany

**Keywords:** Unplanned hospital admission, Palliative care, Specialized outpatient palliative care, Informal caregiver, Family caregiver, Psychological distress

## Abstract

**Background:**

Although the majority of German patients in a palliative state prefer to die at home, the actual place of death is most often a hospital. Unplanned hospital readmissions (UHA) not only contradict most patients’ preferences but also increase the probability of an aggressive end-of-life treatment. As limited knowledge is available which factors contribute to an UHA, the PRePP-project aims to explore predictors related to informal caregivers (IC) as well as medical and structural factors.

**Methods:**

This prospective, observational, mono-centric study will assess structural and medical factors as well as ICs’ psychological burden throughout seven study visits. Starting in April 2021 it will consecutively include 240 patients and their respective IC if available. Standardized measures concerning ICs’ Quality of Life (WHOQOL-BREF), psychological distress (NCCN-Distress Thermometer), anxiety (GAD-7) and depressiveness (PHQ-9) will be assessed. If participants prefer, assessment via phone, browser-based or paper-based will be conducted. Medical records will provide routinely assessed information concerning patient-related characteristics such as gender, age, duration of hospital stay and medical condition. Nurse-reported data will give information on whether hospitalization and death occurred unexpectedly. Data will be progressed pseudonymized. Multivariable regression models will help to identify predictors of the primary endpoint “unplanned hospital admissions”.

**Discussion:**

The PRePP-project is an important prerequisite for a clinical risk assessment of UHAs. Nevertheless, it faces several methodological challenges: as it is a single center study, representativity of results is limited while social desirability might be increased as the study is partly conducted by the treatment team. Furthermore, we anticipated an underrepresentation of highly burdened participants as they might refrain from participation.

**Trial registration:**

This study was retrospectively registered 19 October 2021 at clinicaltrials.gov (NCT05082389). https://clinicaltrials.gov/ct2/show/NCT05082389

## Background

With evolving treatment options in several diseases, prolonged survival at the cost of a more complex end-of-life treatment has been achieved in the recent past. In Germany, the need of adequate palliative care as well as the number of structures providing palliative care has increased [1]. In 2014, more than 33.000 cases were treated in specialized palliative care units, 30.000 in hospices and almost 87.500 cases were reported by specialized outpatient palliative care teams [2].

Between 52 and 94% of German patients prefer end-of-life care at their home [3, 4] while the actual place of death was home in less than one third of deceased persons [5] in 2009. Specialized outpatient palliative care teams (german abbreviation: Spezialisierte Ambulante Palliativversorgung, SAPV) aim to alleviate symptoms and enable incurably ill patients to stay at their familiar surrounding until death by providing sufficient end-of-life care. Evidence suggests various advantages of preventing patients dying in the hospital. Gomes et al. report that home palliative care increases the chance of dying at home and reduces symptom burden in particular for patients with cancer [6]. Quality of life tends to be higher in patients dying at home [7]. In addition, optimal care by informal caregivers (IC) leads to greater satisfaction and less grief on the part of the relatives [8]. Yet there is a limited understanding of comprehensive costs of palliative care [9], caring at home might relieve financial burden off the health care system as inpatient care accounts for more than 70% of costs [10] during a palliative phase.

Unplanned hospital admissions (UHAs) decrease the probability to die at home [11] and were identified as a factor of overly aggressive treatment near the end of life [12]. UHAs occur due to various reasons including physical, psychological and social factors as well as characteristics of professional support [6, 13]. According to Gamblin and colleagues [14], UHAs occur more frequently in patients being released from palliative care unit (compared to medical oncology ward), which is attributed to a more complex burden of symptoms. Evidence derived from lung cancer patients of a single center suggest UHAs are the result of cancer-related symptoms rather than treatment toxicities [15]. Gamblin et al. illustrated that living alone at home or the presence of at least one child at home were associated with death in hospital [14] while proposing that financial precariousness, exhausted or ill caregiver might contribute to readmission. Furthermore, UHAs caused by a medical indication can also be altered by the psychosocial factors at the patient’s place of residence [16]. A post-discharge palliative care consultation was associated with a significantly lower incidence of UHA [14].

The frequency of UHA as well as the place of death varies internationally [17]. These discrepancies can be explained both by different health care systems with a varying extent of hospice and palliative services [18] and by discrepancies of insurance coverage policies for hospital and palliative care [19–21]. The consensus of experts of the EAPC (European Association of Palliative Care) recommends a minimum of one SAPV-Team per 100.000 inhabitants, while in Germany only 0.22 SAPV-team per 100.000 inhabitants is available [22] with huge differences between federal states. In addition to specialized palliative care, there are further structures available in Germany: Alongside general practitioners and specialists, mobile nursing services and voluntary hospice organizations are involved in the care of palliative patients.

However, a major part of the care is provided by informal caregivers. This includes all family members, friends or neighbors who are involved in the care of a person with health-related restrictions usually without receiving payment [23]. In Germany, up to 4.7 million informal caregivers take care of a frail relatives [24], yet there is no structured evidence available how many of them support terminal-ill patients.

Yet recent research suggests that including informal caregivers in the disease management may improve patients’ outcomes [25], it might bear several burdens on the informal-caregiver [26]. These include physical symptoms such as decreased immune functioning, fatigue or sleep disturbance as well as high psychological distress, anxiety and depression [27, 28]. It remains unclear too which extent these burdens contribute to UHA as caregivers, often old or frail him-/herself, might be overwhelmed or too exhausted to provide sufficient care at home.

Overall, there is scarce evidence on factors associated with UHAs in palliative outpatients. It seems of utmost importance to prevent UHAs in order to meet patients' preferred place of death, alter support of the professional system according to the informal caregivers needs and provide economic responsible options for the treating hospital. This project will explore the role of medical, structural as well as caregiver-associated factors as predictors for UHA. It is an important prerequisite for both, clinical implications as well as further research activities.

## Methods/design

### Study design and setting

Starting in April 2021, the PRePP-project is a prospective, observational, longitudinal, single-arm, single center study to identify structural and medical factors as well as those related to a patient’s informal caregiver which predict (unplanned) hospital readmission.

Primary study aim is to explore structural, medical as well as IC-reported factors associated with UHAs. Secondary goals include the description of ICs’ psychological distress and supportive care needs.

This study is performed at the SAPV-team of a large tertiary cancer center treating about 300–330 palliative patients a year.

### Participants and recruitment

The PRePP-project aims to include two samples of participants, patients and their respective care providers. It aims to include consecutively 240 patients and, if present and consenting, their respective primary IC within 18 months.

Eligible patients who are adult (> 17 years), suffer from an advanced, life-limiting disease and are treated by the SAPV-team of our institution will be included.

The primary non-professional caregiver is defined as the adult person that provides most time supporting the patient regardless of being biological related or non-related. ICs can participate if they provide informed consent, are mentally and linguistically able to provide answers independently. IC-reported data can only be collected for patients with consenting ICs. Due to this condition the exact number of IC-participants cannot be predicted.

### Study visits and data assessment

Data is obtained from three sources: (1) IC-reported, (2) medical-record based and (3) nurse-reported.

**Fig. 1:**
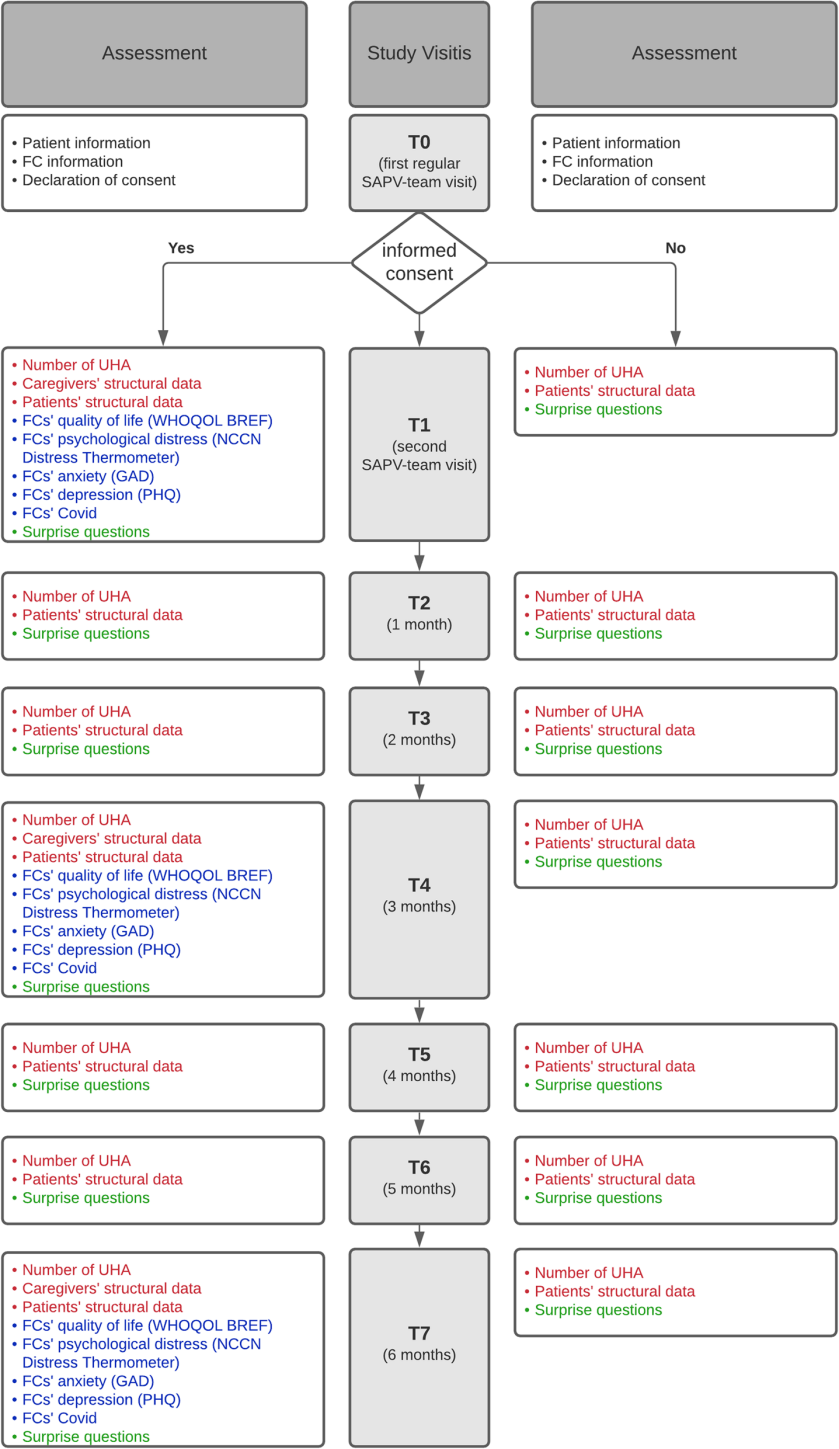
Timeline of study visits and source of data. Data source red = medical record; blue = family care-giver; green = nursing practitioner

Data of consenting ICs will be obtained three times. Medical-record based and nurse reported data will be obtained seven times. Detailed timeline of study visits can be found in Fig. 1.

After informed consent, which will be obtained at the first regular visit of the SAPV-team (T0), the initial visit collects data of all three sources via tablet-PCs provided by SAPV-team at second home visit (T1). Two subsequent study visits after three (T4) and six months (T7) obtain IC-reported data remotely either browser-based, by mail (paper–pencil) or via phone by study staff. The monthly (T1-T7) assessment includes data derived from medical records and nurse-report.

If no ICs are available or refuse to participate, only the patient’s medical report, living situation and nurse-reported data will be gathered monthly (T1—T7).

Electronic data is captured applying REDcapp [29, 30].

### Ethics and trial registration

Ethical approval is obtained prior to recruitment from local institutional review board (BO-EK-320072020). According to German law (§34SächsKHG), no study specific informed consent must be obtained from patients to use routinely assessed data from medical records. This trial is registered at clinicaltrials.gov (NCT05082389).

### Study measures

Informal caregivers willing to participate answer standardized measures on several domains.

Quality of Life will be assessed administering the WHOQOL-BREF [31], a 26-items inventory to assess a person’s Quality of Life including the four domains physical health, psychological, social relationships and environment. Higher scores denote higher Quality of Life in this domain.

Psychological distress will be assessed by the NCCN-Distress Thermometer [32] which comprises of a visual analogue scale ranging from zero to ten with higher scores indicating a more severe burden and a checklist of various symptoms. It is extensively used and cut-off data is available for use in an oncological setting [32]. Preliminary, it was developed for use in cancer patients while it might be applicable in other settings as well [33].

Anxiety will be assessed applying the Generalized Anxiety Disorder 7-item scale [34], a seven-items patient-reported questionnaire with sufficient psychometric properties. Scores range from zero to 21 on a single scale with higher scores indicating a higher level of anxiety. Results can be classified in either one of four classes ranging from minimal to severe anxiety symptoms.

Depressivness will be measured administering the Patient Health Questionnaire [35], which comprises of nine items. A single sum score is calculated which ranges from 0 to 27 with higher scores indicating a higher level of burden. Results are categorized in four classes ranging from no depressive symptoms to severe symptomatology.

Due to the possible influence of the current COVID-19 pandemic, four self-developed questions considering IC’s worries regarding an infection, changes in professional support and psychological burden due to preventive measures such as wearing a face mask were administered.

Nurses will answer two surprise questions [36], predicting whether they would be surprised when the patient will die within the next month (first question) and whether an UHA will occur during the next month (second question).

Characteristics of IC such as age, gender, and kind of relation to the patient, duration of daily care, highest education, and employment status will be obtained. Furthermore, the amount and type of professional support such as mobile nursing teams will be assessed.

Medical records will provide routinely assessed information concerning characteristics of patient such as age, gender, diagnosis, duration of previous hospitalization, additional supportive services, number of visits by SAPV, symptoms status, admission (e.g. reasons, initiating person, admitting physician, planned vs. unplanned, which procedures applied).

### Statistical analysis

The primary endpoint "number of unplanned inpatient admissions" is account variable. Univariate Poisson regressions will be used to analyze the association of structural factors and relative-reported factors or structural data with the primary endpoint. With the planned patient number of 240, it is possible to detect changes of 50% (corresponding to rate ratios of 1.5 and greater, given a base rate of 0.85) with a power of 95% if the influence variable is a binary categorical variable with 50% probability of occurrence. For a binary factor with a probability of occurrence of 90%, the detectable rate ratio with 95% power is still 2.0. For continuous, normally distributed influence variables, rate ratios of > 1.25 are detectable with 95% power.

Since a large number of factors are to be tested, the adjustment for multiple testing seems reasonable. According to the explorative approach of the study, the control of the false discovery rate is done by Benjamini–Hochberg procedure. *P* values of the univariate regression analyses that are < 0.05 after adjustment with the Benjamini–Hochberg procedure are considered significant. In the multivariable model, all parameters whose *p* values from the univariate analyses are < 0.1 after adjustment should be analyzed. The *p*-values of the multivariable model will not be adjusted.

## Discussion

The PRePP-projects aims to provide an elaborated understanding of factors predicting hospital readmission in palliative outpatients. As scarce knowledge is available on this issue and little comparable studies are available, we face some practical and operational challenges which future studies could keep in mind.

Representativity and generalizability could be limited as the project is a single-arm and single-center study. This may cause selection bias for certain patients’ and ICs’ characteristics regarding regional and cultural differences. A multicenter study design could reduce this kind of bias.

The different methods of collecting ICs’ data could induce varying response behavior. Participants acquired by mail or browser-based may feel more anonymous causing more reliable answers, compared to phone surveys. Possible bias due to social desirability while communicating directly with the study staff, could occur. Moreover phone surveys performed by different members of the study team could cause interviewer bias. However we decided to use the opportunity of phone surveys to keep response rate higher, considering elderly participants as not as comfortable using online questionnaires and avoid additional effort for them by returning the mail. As a practical issue we anticipate less participation of highly burdened ICs which could lead to an underestimation of participants' burden.

Concerning the questionnaires used, we face a trade-off between diagnostic accuracy on the one hand while reducing the burden for the participants on the other hand. We decided to administer short instruments such as GAD-7 or PHQ-9 as they use a smaller number of questions and are self-administered compared to structured interviews such as the Structured Clinical Interview for DSM-5 [37]. Furthermore, we aim to describe psychological burden rather than diagnose a psychiatric disease which allows for less sophisticated instruments. This is well in line with other groups performing research in palliative care [38].

As this study was conceptualized during the beginning of the COVID-pandemic there were no standardized questions reagarding ICs concerns about COVID available. Future research should progress on this issue, e.g. by developing standardized instruments or at least performing psychometric analyses on available questions.

Regardless of aforementioned challenges, the data obtained from the PRePP-Project serves as an important prerequisite for the development of a screening tool to predict readmission which could be administered to patients routinely in clinical practice.

## Data Availability

The datasets used and/or analysed during the current study are available from the corresponding author upon reasonable request.

## Abbreviations

EAPC: European Association of Palliative Care
GAD-7: Generalized Anxiety Disorder 7-item scale
IC: Informal caregiver
NCCN: National Comprehensive Cancer Network
NP: Nursing practitioner
PHQ-9: Patient Health Questionnaire 9-item scale
SAPV: Specialized outpatient palliative care (German: Spezialisierte Ambulante Palliativversorgung, abbreviation: SAPV)
T0-T7: Time; indicates study visit
UHA: Unplanned hospital admission
